# Efficient Recovery of Phosphorus from Wastewater Using Calcium-Based Modified Biochar: Removal Performance, Adsorption Mechanism, and Resource Utilization

**DOI:** 10.3390/toxics13100808

**Published:** 2025-09-23

**Authors:** Yihe Qin, Run Yuan, Han Li, Haiming Huang

**Affiliations:** 1 School of Environment and Civil Engineering, Dongguan University of Technology, Dongguan 523808, China; qinyihely@163.com (Y.Q.); qyuanrun@163.com (R.Y.); lihan@dgut.edu.cn (H.L.); 2 School of Earth System Science, Tianjin University, Weijin Road, Tianjin 300072, China

**Keywords:** biochar, phosphates, eggshell, resource utilization, microbial diversity

## Abstract

Phosphorus, a crucial yet nonrenewable resource, is essential for agriculture, life processes, and various industries. In this study, we employed co-pyrolysis of eggshells and peanut shells to prepare calcium-based biochar (EPB) with a high adsorption capacity and ecological non-toxicity, enabling effective phosphorus recovery from wastewater. EPB was characterized via X-ray diffraction, scanning electron microscopy, electron probe microanalysis, and Brunauer–Emmett–Teller analysis. Additionally, its phosphate adsorption characteristics were investigated under varying temperature, pH, and coexisting ion conditions. Phosphate adsorption followed the Langmuir isotherm with a maximum adsorption capacity of 178.08 mg/g, and the kinetics aligned with those of the quasi-second-order kinetic model. Phosphate adsorption by EPB was driven by electrostatic attraction and chemical precipitation. Moreover, we investigated the effects of phosphorus-enriched biochar on the growth and development of tobacco and soil microbial communities. Phosphorus-enriched biochar increased organic and inorganic phosphorus levels and promoted tobacco growth compared with conventional fertilizers. Phosphorus-enriched biochar reshaped tobacco rhizosphere microbial communities, promoting beneficial taxa, such as *Nitrospira*. Structural equation analysis showed that EPB enhanced microbial alpha diversity and key microbial communities, improving phosphorus availability and tobacco growth and development. Conclusively, this study provides a theoretical reference for phosphorus-containing wastewater treatment and reuse.

## 1. Introduction

Phosphorus is an essential nutrient for plants and other life forms [1]. Countries rely on phosphate fertilizers extracted from phosphate rocks to maintain crop yields and ensure food security [2,3]. However, high losses and low utilization efficiency in the phosphorus supply chain raised concerns regarding its shortages. Regarding supply, ores with a high phosphorus content are limited, although global phosphorus reserves are estimated to last for 300 years at current mining rates, with China, the United States, and Russia projected to deplete their reserves within 40 years [4]. Additionally, the ratio of phosphorus intake from food to the phosphorus required for growing food is extremely low, at only 5–10% [5]. Indiscriminate use of phosphate fertilizers and inadequate wastewater treatment are the primary sources of phosphorus pollution in aquatic environments [6,7]. Phosphorus pollution in water bodies increases the risk of toxic algal blooms and dead coastal zones and poses a threat to human health and ecological security [8]. Preventing and capturing phosphorus losses before they cause environmental harm, and recycling them into agricultural production to reduce dependence on phosphate ore, is a “win–win” strategy for reducing national phosphorus vulnerability. Therefore, it is of great research significance to properly address the issue of phosphorus pollution and form phosphorus resource recycling.

Compared with those of other phosphorus treatment processes, such as chemical (precipitation, crystallization, anion exchange, and adsorption) [9,10,11], biological (enhanced biological phosphorus removal and artificial wetlands) [12,13], and physical processes (microfiltration, reverse osmosis, electrodialysis, and magnetic separation) [14,15,16], adsorption has the advantages of low cost, high efficiency, selectivity, and simple operation [17]. Recently, the application of porous biochar as an adsorbent has attracted considerable research interest because biochars can be prepared from waste biomass using a wide range of inexpensive raw materials such as agricultural waste, compostable materials, and renewable natural resources [18,19]. Notably, phosphate-saturated biochar can be used as a phosphate fertilizer, enabling the treatment of phosphate-containing wastewater and the utilization of phosphate resources, while also contributing to carbon reduction goals in the context of carbon peaking and neutrality. Biochar obtained through high-temperature activated pyrolysis can also be used in soil amendment with advantages such as improved soil health, phosphorus availability, and agricultural ecosystem productivity. However, biochar directly prepared from biomass has a negatively charged surface and limited adsorption capacity. For example, buckwheat shell charcoal can negatively adsorb inorganic phosphorus. To address this issue, commonly used metal cations (such as Ca^2+^, Fe^3+^, Mg^2+^, Al^3+^, and La^2+^) are used to modify biochar to increase its affinity for phosphorus [20,21,22,23]. Calcium, as a modifier, has the advantages of ecological non-toxicity and abundance [24].

Eggshells are a waste rich in calcium, containing more than 94% CaCO_3_, and are a cost-effective source of Ca^2+^. Annually, 8500,000 t of eggshell waste are generated globally.

Importantly, the preparation of various calcium-modified biochar by co-pyrolysis of natural calcium-containing eggshells and carbon materials can avoid the use of calcium-containing chemical agents to reduce production costs and increase adsorption performance. Liu prepared Ca-biochar composites via pyrolysis of calcium-modified peanut shell at 400 °C and found that the Ca-biochar’s adsorption capacity reached 150.2 mg/g, achieving high phosphorus removal efficiency in water [25]. Additionally, Li Jing et al. reported adsorption capacities of 154.18 mg/g and 129.03 mg/g using activated sludge-based biochar loaded with calcium from egg and oyster shells, respectively [26]. Additionally, calcium is a nutrient needed by plants. Moreover, phosphorus-adsorbed biochar contains calcium and phosphorus and has the potential for soil phosphorus supplementation. Quisperima et al. reported that Ca-biochar composites prepared via high-temperature pyrolysis of potato peels and eggshells had an adsorption capacity of up to 174.8 mg/g and effectively promoted plant growth [27]. However, studies on the mechanisms underlying the responses of soil microorganisms and p-pools following biochar application are limited. Considering that no gaseous phosphorus compounds are detected in the atmosphere, it can be concluded that phosphorus undergoes a typical sedimentary cycle that takes place mainly between soil, plants, and microorganisms. Soil microorganisms participate in the dissolution of inorganic phosphorus compounds and the mineralization of organophosphides, as well as in the fixation of administrable phosphorus. As soil nutrient cycling is largely determined by microbial activity, an in-depth understanding of microbial responses to phosphorus-rich biochar can support soil fertility, plant health, and ecosystem impacts and expand our understanding of the potential of biochar-based phosphate adsorbents as phosphate fertilizers. Although there is limited research on the adsorption effect of calcium-modified peanut shell biochar on inorganic phosphorus and the effect of peanut shell charcoal on soil improvement, some studies confirmed that peanut shell biochar has a good effect on the adsorption of pollutants such as Cu, Ni, Pb, methyl orange, and other pollutants after modification by metal salts, acid, and alkali.

Here, peanut shells were selected as modified biochar and eggshells as calcium sources to investigate the phosphate adsorption characteristics of eggshell-based biochar (EPB) and its application in agricultural fertilizers. Based on the preparation mechanism of EPB, the factors influencing its phosphate adsorption mechanism and the underlying adsorption mechanism were examined. Additionally, the effects of phosphate-loaded EPB (EPB-P) on tobacco rhizosphere microorganisms and phosphorus conversion were investigated. Overall, it is anticipated that this study will provide a scientific basis for the phosphate treatment of wastewater and phosphorus recovery from phosphorus-enriched biochar.

## 2. Materials and Methods

### 2.1. Test Materials

The peanut shells used here were obtained from Henan Province, washed, dried, and sieved through a 30 mesh (0.6 mm) sieve for future use. Eggshells were collected from local restaurants, washed, dried, and sieved through a 200 mesh (0.074 mm). Eggshells and peanut shells were dried in an oven at 60 °C. Analytical grade KH_2_PO_4_, H_2_SO_4_, NaCl, NaNO_3_, Na_2_SO_4_, NaHCO_3_, NaOH, Ca (H_2_PO_4_)_2_, NH_4_NO_3_, and K_2_SO_4_ were purchased from McLean Co., Ltd. (London, UK).

### 2.2. Test Methods

#### 2.2.1. Preparation of Adsorbents

Egg and peanut shells were mixed in weight ratios of 0:1 (PB), 1:5 (EPB1:5), 1:3 (EPB1:3), 1:2 (EPB1:2), and 1:1 (EPB1:1), and then ball-milled for 30 min. Thereafter, the mixture was pyrolyzed in a horizontal tube furnace at temperatures of 500, 600, 700, 800, and 900 °C under a nitrogen atmosphere for 2 h (heating rate of 5 °C/min). Additionally, a sample (EB) containing only eggshells was prepared under the same conditions at 800 °C. After pyrolysis, the sample was sieved to a <150 μm particle size.

#### 2.2.2. Adsorption Test

Adsorption experiments were conducted by varying the contact times, phosphate pH, temperatures, and anion types. After collecting samples at designated intervals, the mixture was immediately filtered through a 0.45 μm filter, and the residual phosphate concentration was determined using ammonium molybdate spectrophotometry to assess phosphate adsorption kinetics on biochar and establish adsorption isotherms. Notably, the phosphate solution was first prepared as a stock solution (500 mg/L) and then diluted to obtain different concentrations. NaOH (0.1–5 mol/L) and H_2_SO_4_ (0.1–1 mol/L) solutions were used to adjust the pH of the phosphate solution. Phosphate levels were determined using the ammonium molybdate spectrophotometric method (Jinghua721, Shanghai, China) at a wavelength of 700 nm (Chinese National Standard GB 11893–89) [28]. Specifically, the experiment was conducted in a shaker (LichenTHZ-82, Shanghai, China) and oscillated at 190 rpm. The experimental parameters (C_0_: initial concentration of phosphate solution for each group, V: volume of phosphate solution, m: biochar mass, and t: oscillation time) were as follows:

(1) Adsorption kinetics: C_0_ = 200 mg/L; V = 0.05 L; m = 0.05 g; pH = 2.00; t = 0–720 min; temperature = 298 K.
(2) pH: initial concentration C_0_ = 200 mg/L; a total of 11 pH treatments (pH: 2.00–13.00); V = 0.05 L; m = 0.05 g; t = 720 min; temperature = 298 K.
(3) Temperature: initial concentration C_0_ = 200 mg/L; temperature = 288, 298, 308, and 318 K; V = 0.05 L; m = 0.05 g; t = 720 min; temperature = 298 K; pH = 2.00.
(4) Adsorption isotherm: C_0_ = 50–350 mg/L; V = 0.05 L; m = 0.05 g; t = 720 min; temperature = 298 K; pH = 2.00.
(5) Anion types: NO_3_^−^, Cl^−^, HCO_3_^−^, SO_4_^2−^ (20–100 mmol/L); C_0_ = 200 mg/L; V = 0.05 L; m = 0.05 g; t = 720 min; temperature = 298 K; pH = 2.00.
(6) Biochar produced under different pyrolysis temperatures and proportions of eggshell addition: C_0_ = 200 mg/L; V = 0.05 L; m = 0.05 g; t = 720 min; temperature = 298 K, pH = 2.00.

The adsorption capacity $\left(q_{t}\right)$ and the equilibrium concentration of phosphate ($q_{e}$) were calculated as follows [26,27]:

(1) $$q_{e}=\frac{{(c}_{0}-c_{e})V}{m}$$

(2) $$q_{t}=\frac{{(c}_{0}-c_{t})V}{m}$$

where $c_{t}$ is the concentration of phosphate in the solution at moment *t*.

Adsorption kinetics were fitted with pseudo-first-order, pseudo-second-order, Elovich, and intraparticle diffusion models. Equilibrium data were fitted using the Langmuir, Freundlich, and Temkin isotherm models (Text S1). The intraparticle diffusion models were expressed in linear forms. Except for intraparticle diffusion models, other models were expressed in nonlinear forms. All experiments had three repetitions, and the average results were reported, with the standard deviations represented by the error bars in the figures. Experimental data were analyzed using the software Origin 2021.

#### 2.2.3. Potted Plant Experiment

A potted plant experiment was conducted in Jia County, Henan Province, China, from May to August, 2024. Basic soil fertility was tested in a plastic pot (top diameter = 52 cm, bottom diameter = 46 cm, height = 33 cm). The test soil, sandy loam in texture, was collected on-site. Briefly, the tested soil was crushed, mixed, and sieved through a 5 mm sieve before the experiment. Each pot contained 35 kg of soil and one tobacco plant, with five replicates (pots) per treatment group. Before the experiment, ridges were formed at the experimental site with a row spacing of 120 × 50 cm. Thereafter, the pots were placed in the ridges and ditches, which were then sealed with soil, ensuring that the top of the plastic pots was level with the surface of the experimental site. Three experimental treatments were set up, as shown in Table 1. The fertilizers applied to each treatment were mixed evenly with the soil within the pots. Tobacco seedlings were transplanted on 2 May 2024. Subsequently, the foot and top leaves were pruned at 70 and 74 days post-transplantation, respectively. Other management measures were consistent with those used for high-quality tobacco cultivation in Henan.

#### 2.2.4. Soil Physical and Chemical Properties and Growth and Development of Tobacco Plants

After 30, 60, and 90 days of transplantation, soil samples were collected from the pots and screened to remove visible animal and plant residues, stones, bricks, and other debris. Thereafter, the soil samples were used to determine various indicators of soil physical and chemical properties. Additionally, plant samples were collected to measure tobacco height, stem circumference, effective leaf number, maximum leaf length, and leaf width.

Soil pH was measured using a pH meter, and the ratio of water to soil was 2.5:1. Available nitrogen (AN), available phosphorus (AP), and available potassium (AK) were detected using alkaline hydrolysis diffusion, molybdenum blue colorimetry, and flame photometer methods, respectively [29]. Additionally, the ammonium nitrogen was colorimetrically modified using indigo phenol blue, and the nitrate nitrogen was analyzed at a wavelength of 625 nm using ultraviolet spectrophotometry (Shanghai Jinghua721, China).

Components, such as phosphate (Ca_2_-P), octacalcium phosphate (Ca_8_-P), aluminum phosphate (Al-P), iron phosphate (Fe-P), occluded phosphate (O-P), and hydroxyapatite (Ca10-P), were extracted using continuous extraction, centrifugation, and filtration with solutions of NaHCO_3_ (0.25 mol/L), NH_4_AC (0.5 mol/L), NH_4_F (0.5 mol/L), NaOH (0.1 mol/L)-Na_2_CO_3_ (0.1 mol/L), sodium citrate (0.3 mol/L)-NaOH (0.5 mol/L), and H_2_SO_4_ (0.5 mol/L), respectively. Notably, the phosphorus contents in these solutions were identified as corresponding forms of inorganic phosphorus components. Organic phosphorus components were classified into labile organic phosphorus (LOP), moderately labile organic phosphorus (MLOP), moderately resistant organic phosphorus (MROP), and highly resistant organic phosphorus (HROP), using the Bowman–Cole method [30].

#### 2.2.5. Characterization

Briefly, the crystal forms of different biochars (EB, EPB1:5, EPB1:3, EPB1:2, EPB1:1, PB) were analyzed using the X-ray diffraction (XRD, Ultimate IV, Japanese science) at a scanning speed of 2°/min. XRD data were analyzed using the X’pert HighScore software 6.0 package and the International Centre for Diffraction Data PDF Database (https://icdd.socolar.com/rdweb, accessed on 11 October 2024) (PDF: 44-1481) [31]. Importantly, the microcrystalline dimensions of EPB1:5, EPB1:3, EPB1:2, and EPB1:1 were based on the FWHM (half-peak full width) of CaCO_3_ (2θ = 33°) and were calculated using the Scherrer Equation (3):

*D = Kλ/βcosθ/* (3)

where *D*, *β*, *λ*, *θ*, and *K* are the crystallite size, FWHM of the selected diffraction peak, wavelength of X-ray radiation, the Bragg angle (in radians), and the shape factor (typically taken as 0.89), respectively.

Additionally, the surface functional groups of EB, EPB1:5, EPB1:3, EPB1:2, EPB1:1, and PB particles were characterized using Fourier-transform infrared spectroscopy (FTIR, Nicolet Nexus 670; Thermo Fisher Scientific, Waltham, MA, USA). Briefly, the sample (1 mg) was mixed with 100 mg of KBr powder, and the FTIR spectrum was recorded in the range of 500–4000 cm. Moreover, the binding behavior between the inorganic phosphorus and EPB1:1 was evaluated using X-ray photoelectron spectroscopy (XPS, Thermo Escalab 250Xi; Thermo Fisher Scientific). The scanning step size of the full scan and that of the narrow scan were 1.0 eV. The thermal decomposition behavior of the adhesive particles containing cold-bonded particles was assessed using thermogravimetric analysis (TGA, SETSYS Evo; SETARAM Instrumentation, Caluire-et-Cuire, France). The standard operating conditions for thermal analysis included a temperature range of 40–1000 °C in a nitrogen atmosphere, with a slope of 10 °C/min. A portable pH meter (PHC10101; Hach Company, Loveland, CO, USA) was used to measure pH. Moreover, the morphology of EPB1:1 was analyzed using scanning electron microscopy (SEM, Hitachi Regulus8100, Tokyo, Japan). Furthermore, the crystal characteristics of Eb1:1 were analyzed using a transmission electron microscope (TEM, FEI Tecnai F20, New York, NY, USA).

#### 2.2.6. Density Functional Theory Calculation Method

Density functional theory (DFT) was used to calculate the binding energy between phosphate and calcium to further verify the adsorption mechanism. The graph was constructed using GaussView 6.0 (Gaussian Inc., Wallingford, CT, USA), and DFT calculations were performed using Gaussian 09 based on B3LYP/6-31G(d,p). The equilibrium geometry of the system before and after adsorption was optimized, and a vibration analysis was conducted. Thereafter, a stable structure with minimum potential energy was obtained.

#### 2.2.7. S rDNA Sequencing

The 16S rRNA sequencing was performed on soil microorganisms before transplantation for the three different fertilizer treatments (CK, BF, NP, CGP, and CGP-P). Briefly, three samples were collected from each group, immediately frozen in liquid nitrogen, and stored at −80 °C until analysis. The variable V4 region was amplified using PCR with 515F and 806R primers labelled with barcodes. Each reaction mixture contained 15 µL Phusion High Fidelity PCR Master Mix (New England Biolabs, Ipswich, MA, USA), 0.2 µM primers, and 10 ng of genomic DNA template. The PCR conditions were as follows: initial denaturation at 98 °C for 1 min, followed by 30 cycles at 98 °C for 10 s, 50 °C for 30 s, and 72 °C for 30 s, and a final extension at 72 °C for 5 min. Magnetic bead purification was performed on the PCR products, which were then pooled in equal proportions based on concentration. The pooled samples were thoroughly mixed, assessed for quality, and the target band was subsequently recovered. Thereafter, a sequencing library was constructed, quantified using Qubit and qPCR, and sequenced. Details of bioinformatics analysis are provided in the Supplementary Materials. Concatenated raw tags were rigorously filtered to obtain high-quality tag data (clean tags) using Fastp software (Version 0.23.1) [32] Thereafter, the obtained tags were further filtered to remove chimeric sequences. Taxonomic annotation of the tag sequences was performed using reference databases: the SILVA database (https://www.arb-silva.de/, accessed on 1 October 2024) for 16S/18S RNA genes and the UNITE database (https://unite.ut.ee/, accessed on 1 October 2024) for ITS regions. Chimeric sequences were identified through comparison and removed to obtain the final effective tags [33].

## 3. Results and Discussion

### 3.1. Preparation Mechanism of EPB

**Figure 1:**
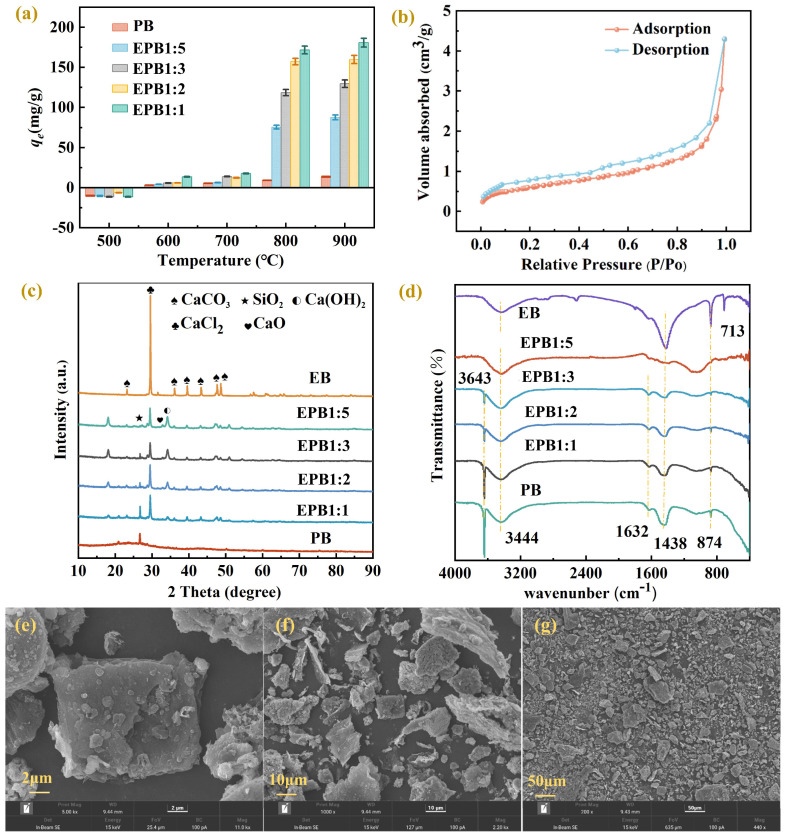
EPB1:5 characterization. (**a**–**c**) Scanning electron microscopy (SEM) images of the outside surface. (**d**) N_2_ adsorption/desorption isotherms. (**e**) The influence of preparation temperature and eggshell addition amount on the adsorption capacity of EPB1:5. (**f**) X−ray diffraction (XRD) patterns of biochar. (**g**) Fourier−transform infrared spectroscopy (FTIR) of biochar.

Pyrolysis temperature crucially impacts the adsorption effect and physical and chemical properties of biochar. Therefore, we investigated the phosphate adsorption efficiency of eggshell-based biochar prepared using pyrolysis at 500, 600, 700, 800, and 900 °C (Figure 1a). Notably, the adsorption effects of biochars prepared via pyrolysis at 800 and 900 °C were significantly higher than those of biochars prepared at 500, 600, and 700 °C, which may be attributed to the insufficient carbonization of biomass and limited surface functional groups due to low temperature. Importantly, the phosphate adsorption capacity increased with increasing temperature. Conversely, low temperature (below 800 °C) caused a decrease in CaO formation from CaCO_3_, which in turn affected phosphate adsorption by EPB [34]. Additionally, the phosphate adsorption effect of calcium-based biochar was higher than that of unmodified biochar, indicating that calcium positively affected phosphate adsorption. Among the treatments, the EPB1:1 group showed the highest adsorption effect. Moreover, no significant differences were observed in the phosphate adsorption effects of EPB1:1 prepared at pyrolysis temperatures of 800 °C (171.65 mg/kg) and 900 °C (180.77 mg/kg). Therefore, considering the energy consumption problem, EPB1:1 (pyrolysis temperature: 800 °C) was selected for subsequent adsorption experiments in tobacco grown in pots. A comparison of the phosphate adsorption capacity of calcium-based biochar with those in the literature (Table S1) showed that the eggshell-based biochar in this study had good adsorption capacity for phosphate.

**Figure 2:**
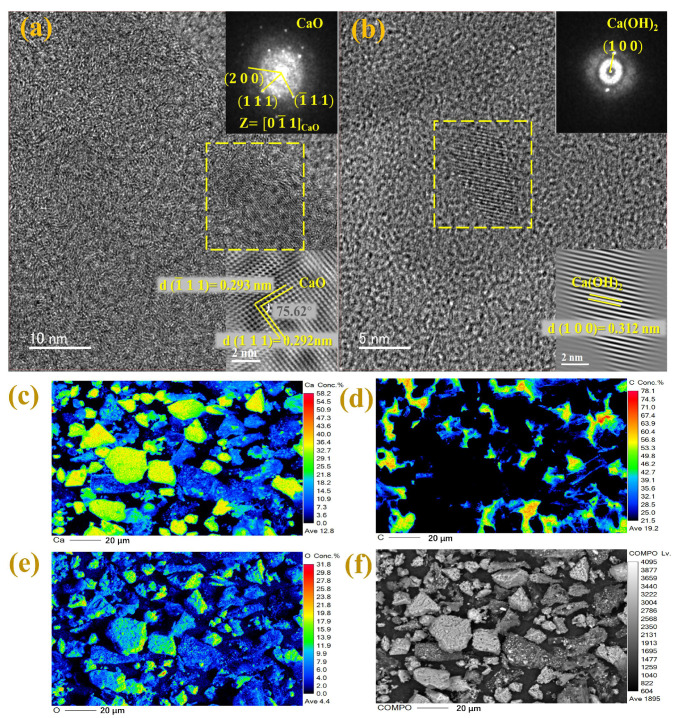
Transmission electron microscopy (TEM) images of EPB1:1 (**a**,**b**) and EPMA images of EPB1:1 (**c**–**f**).

Furthermore, the characteristics of calcium-based biochar were explored using XRD, FTIR, TEM, and EPMA. XRD analysis showed that PB and each calcium-based biochar showed typical peaks (2θ = 22.83°) of a graphite carbon crystal plane (002) (Figure 1c), with the calcium-based biochars showing similar compositions. Additionally, the diffraction peak of EPB1:1 biochar at 29.40° corresponded to CaCl_2_ (PDF: 74–0992); 32.20° corresponded to CaO (PDF: 37–1497); 35.97°, 39.40°, 43.15°, 47.12°, and 48.51° corresponded to CaCO_3_ (PDF: 05–0586); and 18.07° and 34.10° corresponded to Ca(OH)_2_ (PDF: 44–1481) [35]. XRD analysis showed that CaO and Ca(OH)_2_ were present in eggshell-derived biochar, which was consistent with previous findings [36]. Calcium oxide may be produced by the pyrolysis of calcium carbonate in eggshells. Additionally, some calcium oxide can also produce Ca(OH)_2_ with water in the environment [31]. Moreover, the infrared spectra of each biochar and calcium-based biochar at approximately 3643 cm^−1^ could be attributed to the tensile vibration of -OH (Figure 1d), which may be related to the presence of Ca(OH)_2_ [7]. Additionally, the peaks at 1438, 874, and 713 cm^−1^ may be attributed to calcium carbonate tensile vibration [37]. Overall, these results confirm the successful introduction of calcium from eggshells into biochar in the form of calcium oxide and calcium hydroxide. As shown in Figure 2a,b, the lattice spacing of 0.292 and 0.312 nm in the TEM spectra corresponded to the 100 crystal planes of CaO and 111 of Ca(OH)_2_, respectively, indicating that CaO and Ca(OH)_2_ particles were successfully embedded in the biochar. Additionally, the EPMA further confirmed that the charcoal was tightly wrapped by CaO and Ca(OH)_2_ particles (Figure 2c–f). SEM showed that the surface topography of EPB1:1 was irregular and lumpy (Figure 1e–g). BET analysis was performed to examine the specific surface area, pore size, and pore distribution of each calcium-based biochar (thermal decomposition temperature: 800 °C) (Table 2). Notably, the specific surface area and average pore size of calcium-based biochar showed a gradual decrease with increasing addition of eggshells, which was contrary to the findings of Liu et al. [36]. The differences in results could be attributed to differences in the properties of biochar materials. For example, the specific surface area of peanut shell biochar (94.30 m^2^/g) used in this study was larger than that of straw biochar (specific surface area: 7.82 m^2^/g) used in the previous study [38]. Additionally, the CaO generated by the high-temperature pyrolysis process of CaCO_3_ may clog the voids of biochar. Therefore, the specific surface area and average pore size of calcium-based biochar may decrease gradually with increased addition of eggshells. According to the IUPAC classification, the N_2_ isothermal desorption curve of EPB1:1 was a type IV isotherm (Figure 1b), and the average pore size of EPB1:1 was 12.13 nm, making it a potential mesoporous adsorbent. Additionally, the calculation of the grain size of each calcium-based biochar further indicates that the grain size of each calcium-based biochar decreased gradually with increasing addition of eggshell, which may be due to the co-pyrolysis of eggshells and peanut shells to form fine second-phase particles (CaO), thereby preventing further grain growth through Zener pinning [39].

### 3.2. Phosphate Adsorption Performance of EPB

#### 3.2.1. Adsorption Kinetics

**Figure 3:**
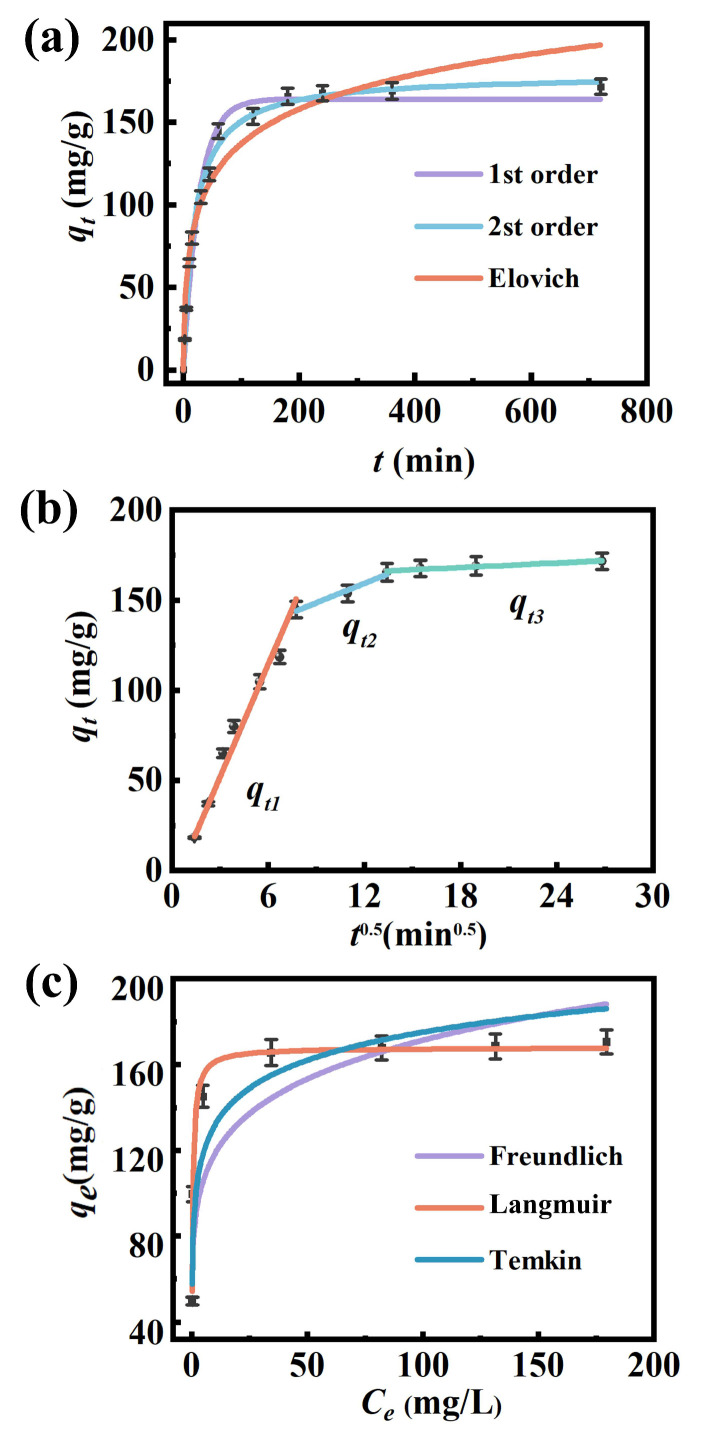
(**a**) Adsorption kinetics fitted using pseudo-first-order, pseudo-second-order, and Elovich models; (**b**) intraparticle diffusion model. (**c**) Adsorption isotherms fitted using Langmuir, Freundlich, Langmuir–Freundlich, and Temkin models.

In this study, we examined the adsorption kinetics of the calcium-based biochar EPB1:1 to phosphate and found that phosphate adsorption by EPB1:1 reached equilibrium in approximately 180 min, at which time the removal rate was 83.88% (Figure 3a). Additionally, the quasi-first-order, quasi-second-order, and Elovich models were used for kinetic analysis, and the corresponding parameters are shown in Table 3. Importantly, the quasi-second-order model (R^2^ = 0.995) can describe the phosphate adsorption behavior of EPB1:1 well. Overall, these results indicate that phosphate adsorption by EPB1:1 included not only physical processes (pore filling and electrostatic attraction) but also chemical processes such as interaction with phosphate, surface precipitation, and ligand exchange [34]. Additionally, the dynamically fitted curve can be divided into three distinct linear regions (Figure 3b and Table S2). In the first stage, phosphate is rapidly adsorbed to the outer surface of EPB1:1. A lower slope was exhibited in the second stage than that in the first stage, indicating that the reaction rate was reduced in the second stage and the adsorption rate was controlled by pore diffusion. Additionally, the line does not pass through the origin, indicating that the adsorption of phosphate by EPB1:1 is mainly controlled by intraparticle and thin film diffusions [40].

#### 3.2.2. Adsorption Isotherm

Figure 3c shows the adsorption isotherm of EPB1:1 for phosphate. The adsorption capacity increased with the initial increase in the concentration of phosphate. At low initial phosphate concentrations, the adsorption capacity of phosphate increased rapidly. However, the adsorption capacity gradually stabilized following an increase in the initial concentration of phosphate, which could be attributed to the gradual occupation of the active site on the surface of EPB1:1, resulting in saturation of the adsorption site. Further fitting of the adsorption isotherms (Table 4) showed that the coefficients of determination of the Langmuir, Freundlich, and Temkin models were 0.9709, 0.7895, and 0.8907, respectively, indicating that the Langmuir model had the best fit (adsorption capacity could reach 167.97 mg/g). Overall, these results indicate that the adsorption of phosphate on EPB1:1 is mainly monolayer, involving chemical precipitation or hydrogen bonding.

#### 3.2.3. Factors Affecting Adsorption

**Figure 4:**
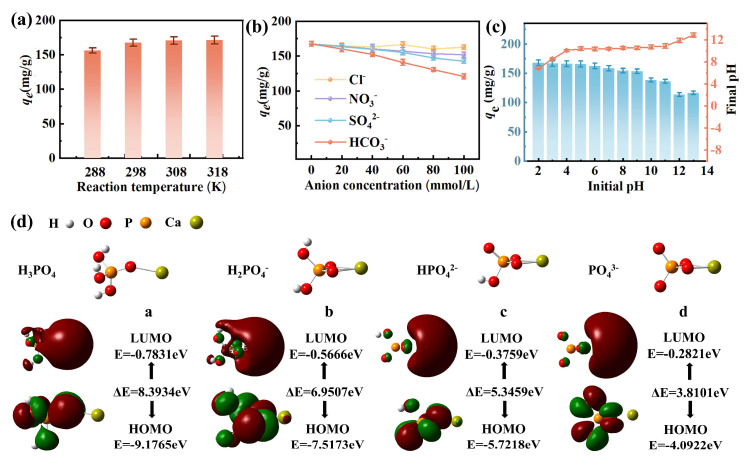
Effects of temperature (**a**), ion type (**b**), and initial pH (**c**) on the adsorption characteristics of phosphate by EPB1:1. Density functional theory (DFT) calculations of different forms of phosphate and Ca ions (**d**).

The phosphate adsorption capacity of EPB1:1 increased with increasing temperature (Figure 4a). Therefore, we calculated the thermodynamic parameters of the adsorption process at 288, 298, 308, and 318 K (Table S3). Notably, ΔG° was negative (ranged from −4.43 to −3.03 kJ/mol), indicating that phosphate adsorption by EPB1:1 was spontaneous and thermodynamically favorable. Additionally, the positive ΔH° value (10.25 kJ/mol) indicated that the adsorption process was endothermic [41]. Moreover, the positive ΔS° value (46.83 J/mol·k) confirmed the strong affinity between EPB1:1 and phosphate and that the randomness of the solid/liquid interface increased during adsorption. Furthermore, the adsorption performance of EPB1:1 on phosphate in the presence of anions (Cl^−^, NO_3_^−^, SO_4_^2−^, HCO_3_^2−^) was examined (Figure 4b). Importantly, the influence of each ion on the adsorption characteristics was in the order of HCO_3_^2−^ > SO_4_^2−^ > NO_3_^−^ > Cl^−^, with HCO_3_^2−^ exerting the strongest inhibitory effect on phosphate adsorption by EPB1:1.

As shown in Figure 4c, the sorbent exhibited good phosphorus removal efficiency over a wide pH range (2.00–9.00). FTIR, XPS, and XRD showed that Ca(II) and phosphate underwent chemical reactions in calcium-based biochar. Therefore, we examined the reaction mechanism of phosphate and Ca(II) using the DFT calculation. Additionally, the morphology of phosphorus was affected by the pH of the solution. Phosphorus exists as H_3_PO_4_ at pH < 2.10 (pKa1 = 2.1), H_2_PO_4_^−^ at pH of 2.10–7.20 (pKa2 = 7.2), HPO_4_^2−^ at pH of 7.20–12.30 (pKa3 = 12.30), and PO_4_^3−^ at pH > 12.30. According to the ion species results of phosphate at different pH conditions, H_3_PO_4_, H_2_PO^4−^, HPO_4_^2−^, and PO_4_^3−^ were selected as the basic bands for DFT calculation, and the optimized structure of the complexes formed with Ca(II) is shown in Figure 4d. According to frontier molecular orbital theory, the energy gap between the lowest unoccupied molecular orbital (LUMO) and the highest occupied molecular orbital (HOMO) (ΔE = E_LUMO_ − E_HOMO_) indicates the ability of molecules to participate in reactions [31]. As ΔE increases, the molecule becomes more stable. Additionally, the ΔE (3.8101 eV) of PO_4_^3−^ and Ca(II) complex was lower than those of H_3_PO_4_, H_2_PO^4−^, and HPO_4_^2−^ when complexed with Ca(II) (8.3934, 6.9507, and 5.3459 eV, respectively), indicating that PO_4_^3−^ had the weakest binding ability to Ca(II), which was consistent with the effect of pH on calcium-based biochar on phosphate. At pH 12.00 and 13.00, calcium-based biochar had the lowest adsorption capacity for phosphates. This may be attributed to the rapid increase in the concentration of OH^−^, which can compete with phosphorus for adsorption sites. Similar results were obtained in previous studies [42].

### 3.3. Adsorption Mechanism

**Figure 5:**
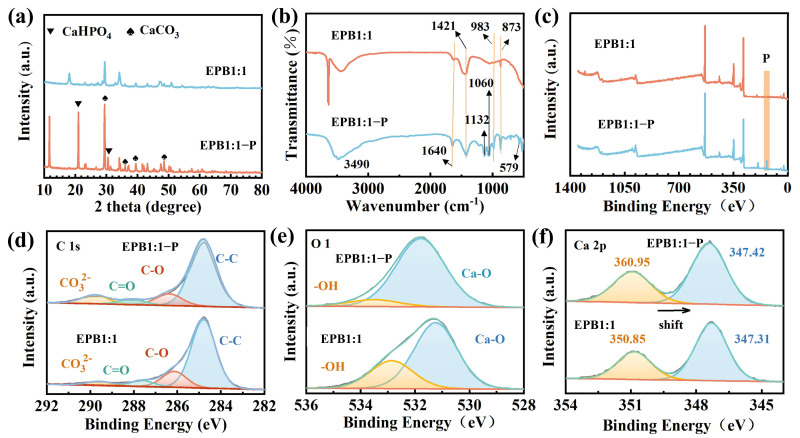
XRD (**a**), FTIR (**b**), XPS total spectra (**c**), C1s (**d**), O1s (**e**), and Ca2p (**f**) of EPB1:1 and EPB1:1-P.

To further elucidate the phosphate adsorption mechanism of EPB1:1, XRD, XPS, and FTIR were used to analyze changes in phase, surface elements, and functional groups of EPB1:1 before and after phosphate adsorption. XRD analysis showed that two new peaks at 21.04° and 30.61° (Figure 5a) were generated after EPB1:1 absorption of phosphate, which was attributed to the response peak of CaHPO4 [36]. Additionally, the presence of this crystal meant that the phosphate may have been removed by precipitation during adsorption. Moreover, the functional group composition of EPB1:1 and EPB1:1-P was analyzed using FTIR (Figure 5b). The characteristic peaks of EPB1:1 included -OH (3490 cm^−1^), C=O/C=C (1640 cm^−1^), and CO_3_^2−^ (1421 cm^−1^, 873 cm^−1^) [43]. However, FTIR spectroscopy of EPB1:1-P indicated the emergence of new response peaks. Among them, the peak at 579 cm^−1^ corresponded to the O-P-O bending vibration and P-O tensile vibration. Additionally, PO_3_ symmetric stretching of HPO_4_^2−^ and P–OH stretching of HPO_4_^2−^ were observed at 1066 cm^−1^ and 983 cm^−1^, respectively. Moreover, the peak at 1132 cm^−1^ corresponded to PO^3−^ stretching [44]. Collectively, these results indicate that phosphorus precipitates on the surface of EPB1:1 in the form of CaHPO_4_.

Figure 5c shows the full XPS spectrum of EPB1:1 and EPB1:1-P. A new P2p peak appeared at 134 eV for EPB1:1-P, indicating that phosphorus was successfully precipitated to the EPB1:1 surface. Additionally, the peaks at ~285 eV, ~348 eV, and ~533 eV in the XPS spectra were attributed to C1s, Ca2p, and O1s, respectively. Moreover, the C1 peaks of EPB1:1 and EPB1:1-P can be attributed to C-C/C-H (284.80 eV), C-OH/C-O-C (286.38 eV), C=O (288.07 eV), and carbonic acid (289.80 eV) (Figure 5d–f). Furthermore, the O1s of EPB1:1 and EPB1:1-P corresponded to -OH and Ca-O. Importantly, the ratio of -OH in EPB1:1-P decreased from 29.69 to 8.99% and the response peak ratio of C-O decreased from 16.97 to 12.91%, which was consistent with the change in the proportion of -OH. Overall, these results indicate that C-OH may be involved in the reaction with phosphate as an electron donor [45]. In EPB1:1-P, a shift in the Ca 2p spectrum was observed (0.109 eV), which was attributed to the electron transfer between the phosphate and Ca. In conclusion, Ca in EPB1:1 mainly exists in the form of CaCO_3_, and the metal ions in EPB1:1 and the phosphate act through electrostatic gravity and hydrogen bonding force during the adsorption process, resulting in the chemical precipitation of CaHPO_4_ to achieve phosphate removal.

### 3.4. Effects of Calcium-Based Biochar on Basic Physical and Chemical Properties of Tobacco Soil and Tobacco Growth and Development

**Figure 6:**
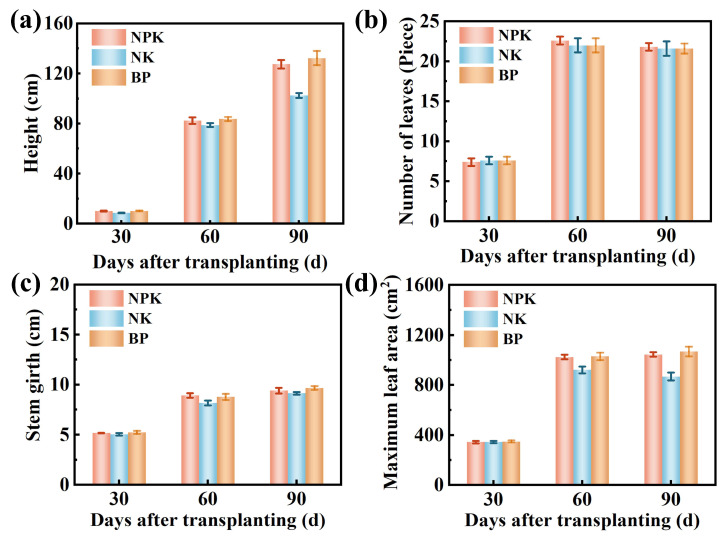
Effects of different fertilization measures on plant height (**a**), effective leaf number (**b**), stem circumference (**c**), and maximum leaf area (**d**) in tobacco plants.

To evaluate the resource utilization potential of EPB1:1-P as a soil phosphorus fertilizer, we examined the growth parameters of tobacco plants and the physicochemical properties of tobacco soil under conventional fertilization (NPK), no phosphorus fertilization (NK), and EPB1:1-P (BP) fertilization (Figure 6a–d). At 60 days post-transplantation, tobacco plants in the NK group had lower height, stem circumference, and maximum leaf area than those in the NPK and BP groups. Additionally, plant heights were 1.24- and 1.29-fold higher in the NPK and BP groups, respectively, than in the NK group at 90 days post-transplantation, indicating phosphorus deficiency may be a key factor limiting plant growth [46]. After 90 days of transplanting, plants in the BP group had slightly higher height, stem circumference, and maximum leaf area than those in the NPK group, and the maximum leaf area in the NPK and BP groups were 1045.32 and 1068.40 cm^2^, respectively. Overall, these results indicate that the effect of EPB1:1-P on tobacco growth and development was comparable to that of conventional chemical fertilizer, highlighting the potential of EPB1:1-P as a phosphorus fertilizer. In addition, the physical photos of tobacco fields are shown in Figure S1.

Table S4 shows the effect of EPB1:1-P on the physicochemical properties of tobacco soil. EPB1:1-P treatment improved the physicochemical properties of tobacco soil, as evidenced by a moderate increase in soil pH from 7.22 to 7.68, which may be caused by biochar ash and surface basic groups [47]. AK, AP, ammonium nitrogen, nitrate nitrogen, and total phosphorus increased by 21.80, 45.61, 59.40, 51.13, and 11.89%, respectively, in the EPB1:1-P group compared with those in the NPK group (Table S4), which could be attributed to the positive effects of calcium-based biochar on soil fertility [48].

### 3.5. Effect of EPB1:1-P on Inorganic and Organic Phosphorus in Tobacco Soil

**Figure 7:**
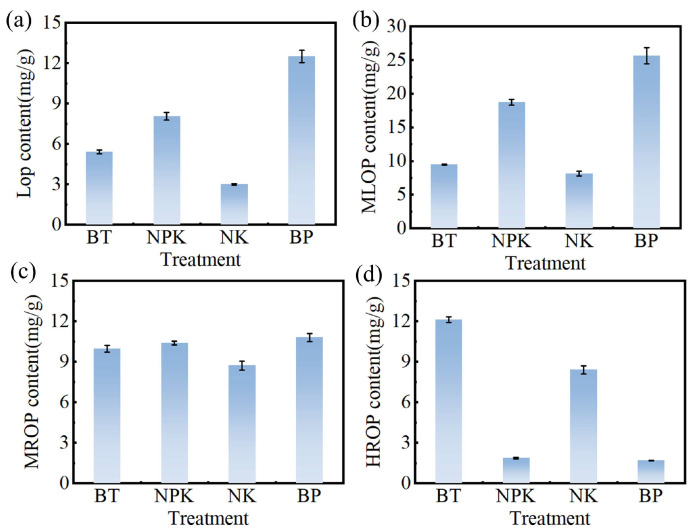
The effects of different fertilization measures on (**a**) labile organic phosphorus (LOP), (**b**) moderately labile organic phosphorus (MLOP), (**c**) moderately resistant organic phosphorus (MROP), and (**d**) highly resistant organic phosphorus (HROP) in tobacco soil.

Figure 7 shows the variations in different forms of soil organic phosphorus. LOP, which is readily mineralized and plant-available, increased significantly with the addition of EPB1:1-P (Figure 7a). During the experimental period, the LOP content was in the order of BP (12.51 mg/kg) > NPK (8.07 mg/kg) > NK (2.99 mg/kg). This pattern may be attributed to the retention of active organic phosphorus derived from biomass during pyrolysis into biochar. During the adsorption processes, phosphorus remains associated with the biochar and can be released into the soil via EPB to enhance LOP levels [49]. MLOP, which is predominantly composed of calcium and magnesium phytates, is relatively susceptible to mineralization. BP treatment significantly increased the MLOP content of the soil by 37.10% compared with NPK treatment (Figure 7b). This increase may be due to enhanced microbial activity and the abundance of phosphorus-solubilizing microorganisms in the BP treatment, which facilitate the dissolution of phosphorus-rich biochar through the secretion of organic acids such as citric acid and gluconic acid [50]. The presence of MROP in the soil has been linked to iron–aluminum oxides and cation exchange capacity (Figure 7c). In this study, BP treatment increased MROP content by 3.88% and 23.86% compared with NPK and NK treatments, respectively. This improvement may be associated with elevated levels of Al-P and Fe-P in the BP-amended soil. Additionally, the addition of EPB1:1-P enhanced soil organic matter, which may contribute to the accumulation of MROP [51,52]. Conversely, HROP is relatively stable and not easily mineralized. Previous studies have indicated that biochar application can promote the conversion of HROP into available phosphorus. In the present study, BP treatment reduced HROP content by 86.04% compared with BT treatment (Figure 7d), indicating that biochar addition facilitates the depletion of this stable phosphorus fraction [52].

**Figure 8:**
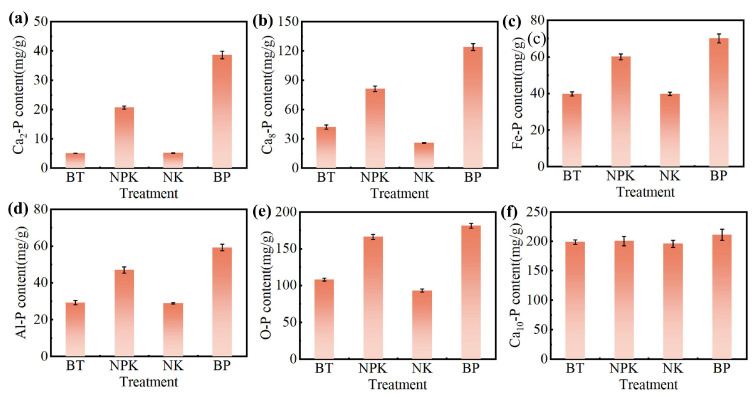
Effects of different fertilization practices on Ca-P (**a**), Ca10-P (**b**), Ca8-P (**c**), O-P (**d**), Fe-P (**e**), and Al-P (**f**) in tobacco soil.

Changes in inorganic phosphorus components under different fertilization treatments are shown in Figure 8a–f. Among the fractions, Ca_2_-p was identified as the most effective source of phosphorus. Ca_2_-p increased by 3.06- and 6.59-fold in the NPK and BP groups, respectively, compared with that in the BT group, which can be attributed to the rapid release of active phosphorus following EPB1:1-P application [53]. Ca_8_-P is considered a slow-release phosphorus source in soil. NPK and BP treatments increased Ca_8_-P content by 0.93- and 1.94-fold, respectively, compared with BT treatment. This may be due to the fact that the potassium dihydrogen phosphate and EPB1:1-P added by NPK and BP treatments contained p and Ca, thereby increasing Ca_8_-P content [54]. Al-P and Fe-P are the slow-release phosphorus sources of soil phosphorus components. Al-P and Fe-P contents increased by 26.23 and 16.60%, respectively, in the BP group compared with those in the NPK group, which may be because EPB1:1 dissolves some hydroxide in the soil solution and raises the soil pH, thereby promoting the adsorption of metal ions by the negatively charged colloid and promoting the formation of Al-P and Fe-P [55,56]. O-P is considered a fixed source of phosphorus in soil, with low activity and difficult to utilize. Compared with that in the BT group, there was an increase in soil O-P content in the NPK and BP groups. Ca_10_-P is a fixed source of phosphorus, mainly composed of certain inorganic insoluble minerals. Generally, biochar addition promoted the conversion of Ca_10_-P to a more active form and enhanced its dissolution by increasing microbial or phosphatase activity, although there were minor variations among the biochar groups [57].

### 3.6. Effect of EPB1:1-P on Tobacco Rhizosphere Microorganisms

**Figure 9:**
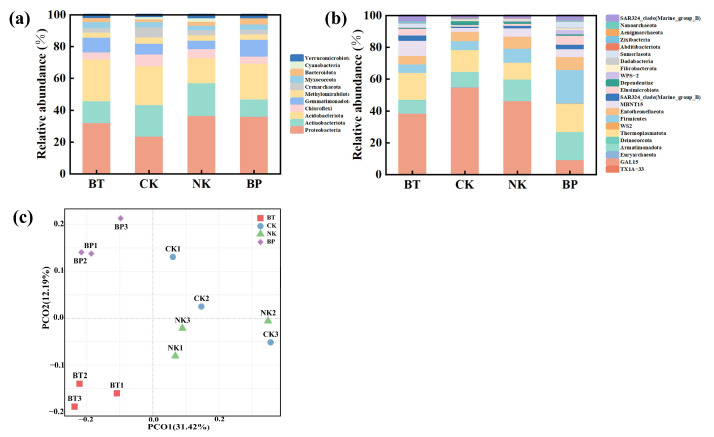
Effects of different fertilization treatments on the microbial community of tobacco rhizosphere soil at phylum (**a**) and genus (**b**) levels. Principal coordinate analysis (PCoA) of microbial community in tobacco rhizosphere under different fertilization treatments (**c**).

The α-diversity index is an important indicator used to quantify the number and relative abundance of species within a community. The Simpson, Shannon, and Chao 1 indices were used to analyze α-diversity. The Simpson index reflects community evenness, while the Shannon and Chao 1 indices assess community diversity [58]. As shown in Table S5, the Chao 1, Shannon, and Simpson indices of the NK, NPK, and BP groups were higher than or equal to those in the BT group. Notably, the Shannon index was highest in the BP group, suggesting that EPB application positively affected the richness and diversity of microbial communities in the tobacco rhizosphere. β-diversity describes the differences in species composition between different communities, and is usually visualized using principal coordinate analysis (PCoA) to reduce the dimensionality of complex microbial data. PCoA shows that the BP samples formed a distinct cluster from the NPK and NK samples (Figure 9c), indicating a significant difference in microbial community structure under different fertilization conditions. Previous studies have shown that an increase in soil carbon positively affects the soil microbial community diversity. Similarly, EPB1:1-P application significantly altered the soil microbial community composition and diversity, which may be due to an increase in soil carbon content and the growth and reproduction of soil microorganisms [59].

At the phylum level, 13, 11, 11, 11, and 9 phyla were dominant (abundance > 1%) in the BT, NPK, BK, and BP groups (Figure 9a). Notably, the most dominant phyla in the BT group before tobacco cultivation were Proteobacteria (25.46%), Acidobacteriota (27.01%), Actinobacteriota (12.80%), and Gemmatimonadota (8.36%) (Figure 9b). A similar trend was observed in the NPK, BK, and BP groups after tobacco cultivation. Specifically, Chloroflexi (4.166.94) replaced Gemmatimonadota as the fourth most abundant phylum, indicating that these four genera may be highly competitive or have specific ecological adaptability under tobacco cultivation ecological environmental conditions, which was consistent with the findings of Wenjing Wang [60]. Compared with those in the NPK group, there was a significant decrease in Cyanobacteria and Verrucomicrobiota abundances and a 49.29% increase in Proteobacteria abundance after the addition of phosphorus-rich biochar. Overall, these results indicate that EPB1:1-P significantly affects the microbial community structure of the tobacco rhizosphere. Proteobacteria are essential for the global carbon, nitrogen, and sulfur cycles, while proteobacteria enrichment can reduce bacterial wilt [61]. EPB1:1-P application indirectly enhanced the resistance of tobacco to bacterial wilt by increasing the abundance of Proteobacteriaceae. At the genus level, the genus with the highest abundance in all treatments was Sphingomonas. Compared with that of BT, the other three treatments showed significant changes in microbial community at the genus level. Specifically, *Subgroup_10*, *Dongia*, and *Allorhizobium*–*Neorhizobium*–*Pararhizobium*–*Rhizobium* all increased to varying degrees. Additionally, *Nitrospira* abundance increased significantly from 0.64% in the NPK group to 1.35% in the BP group. *Nitrospira* abundance was similar in the NPK and NK groups. Nitrospira, a genus of bacteria active in the soil nitrogen cycle, converts nitrates to nitrites, promoting plant growth, which may be responsible for the increased soil nitrate nitrogen content in the BP group. Additionally, soil nitrate nitrogen can increase plant resistance to diseases, such as root rot and bacterial wilt [61]. The main reason is that soil nitrates can promote the induce the activation of signal transduction components such as salicylic acid and polyamines. Therefore, EPB1:1 addition may have increased the abundance of antagonist and nitrogen cycling bacteria, thereby increasing the disease resistance of tobacco and reducing the incidence of diseases, which is beneficial to tobacco growth.

**Figure 10:**
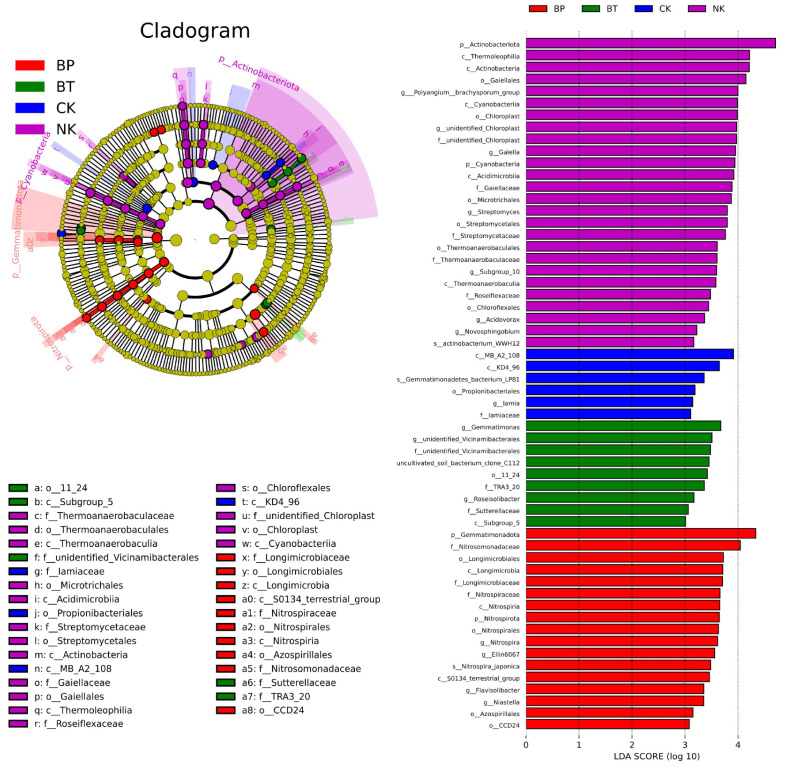
LefSe analysis of microbial community in rhizosphere soil of tobacco plants under different fertilization treatments.

**Figure 11:**
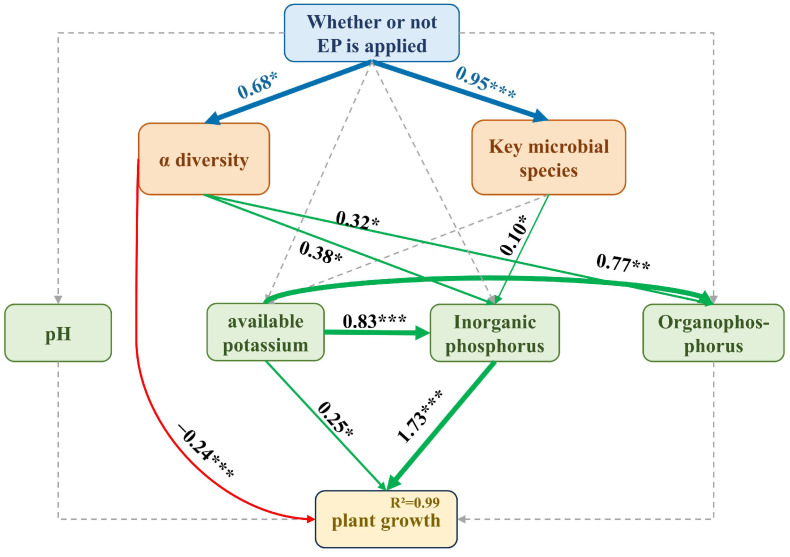
Structural equation analysis of the effect of adding EPB on the growth and development of tobacco plants (“*”: Indicates *p* ≤ 0.05; “**”: Indicates *p* ≤ 0.01; “***”: Indicates *p* ≤ 0.001).

LefSe analysis further identified specific microbial taxa responding to each fertilization treatment (Figure 10). Specifically, 27, 6, 9, and 17 key ASVs exist in BT, CK, NK, and BP, respectively. Further, the structural equations were constructed by combining the key ASVs with soil physicochemical properties and plant growth (Figure 11). EPB1:1-P addition positively affected α-diversity and the abundance of key microbial communities, indirectly positively affected the content of organophosphorus and inorganic phosphorus, and promoted tobacco growth and development.

## 4. Conclusions

In this study, egg and peanut shells were used as raw materials to prepare EPB with high adsorption capacity and ecological non-toxicity. A series of characterization analyses showed that EPB is a potential mesoporous adsorbent. Additionally, the optimal preparation temperature for EPB was 800 °C, at which CaO and Ca(OH)_2_ formed during eggshell pyrolysis were effectively encapsulated by carbon. EPB grain size decreased gradually with increasing addition of eggshells. EPB exhibited the best phosphate adsorption at an eggshell-to-peanut shell ratio of 1:1. Among the models examined for the adsorption experiments, the Langmuir model exhibited good performance (*R*^2^ = 0.995), with a maximum EPB adsorption capacity of 178.08 mg/g. Additionally, phosphate adsorption was endothermic, and the influence of each ion on the adsorption characteristics was in the order of HCO_3_^2−^ > SO_4_^2−^ > NO_3_^−^ > Cl^−^. Adsorption kinetics analysis revealed that the quasi-second-order kinetic model accurately described the process of phosphate adsorption by EPB. Phosphate adsorption by EPB reached equilibrium ~180 min, with a removal rate of 83.88%. Mechanistic analysis showed that phosphates were primarily adsorbed onto the EPB surface through electrostatic attraction, followed by their removal through precipitation as CaP_2_O_6_ and Ca_2_P_2_O_7_ formed with Ca in EPB. In pot experiments, phosphorus-enriched biochar promoted the growth and development of tobacco plants. Moreover, phosphorus-enriched biochar significantly affected tobacco rhizosphere microbial community structure and promoted beneficial microbial taxa such as *Nitrospira*. Structural equation modeling showed that EPB positively affected microbial α-diversity and key microbial communities, which in turn enhanced organic and inorganic phosphorus availability, ultimately promoting tobacco growth and development. Our study provides a theoretical reference for the treatment and utilization of phosphorus-containing wastewater.

## Figures and Tables

**Table 1:** The types and amounts of different fertilizers for each treatment.

Treatment	Organic Material	Chemical Fertilizer		
		NH_4_NO_3_	Ca(H_2_PO_4_)_2_	K_2_SO_4_
		(kg/hm^2^)	(kg/hm^2^)	(kg/hm^2^)
NPK	-	188.57	217.52	366.51
NK	-	188.57	-	366.51
BP	BP1:1-P 1295 kg/hm^2^	188.57	-	366.51

**Table 2:** BET parameters and microcrystalline size of various calcium-based biochars.

Parameter	Surface Area (m^2^/g)	Pore Volume (×10^−2^ cm^3^/g)	Average Pore Size (nm)	Grain Size
EPB1:1	2.19	0.66	12.13	5.97
EPB1:2	1.85	0.47	10.08	20.97
EPB1:3	2.45	0.77	12.65	44.07
EPB1:5	87.3 4	0.14	6.59	77.12
BP	94.30	12.07	5.12	-

**Table 3:** Constants and coefficient of determination for the kinetic models.

Model	Parameter	
Pseudo-first-order model	*q*_e_ (mg·g^−1^)	164.13 ± 3.89
	*k*_1_ (min^−1^)	0.038 ± 0.003
	*R* ^2^	0.980
	*RMSE*	8.77
Pseudo-second-order model	*q*_e_ (mg·g^−1^)	179.08 ± 2.43
	*k*_2_ (g·(mg·min)^−1^)	2.95 × 10^−4^ ± 2.09 × 10^−5^
	*R* ^2^	0.998
	*RMSE*	4.23
Elovich model	α	27.12 ± 9.17
	β	0.033 ± 0.003
	*R* ^2^	0.954
	*RMSE*	13.21

**Table 4:** Isotherm model parameters obtained from adsorption.

Isotherm Model	Parameter	
Freundlich	*K*_F_ (mg^(1−n)^·L^n^/g)	81.45 ± 10.32
	1/*n*	6.193
	*R* ^2^	0.790
Langmuir	*K*_L_ (L/mg)	2.37 ± 0.31
	*RMSE*	6.48
	*Q*_max_ (mg/g)	167.97 ± 6.91
	*R* ^2^	0.97
	*RMSE*	2.61
Temkin	*A* (L/mg)	104.11
	*B* (J·g/mg)	130.88
	*R* ^2^	0.89
	*RMSE*	5.06

## Data Availability

The original data presented in this study are included in the article. Further inquiries can be directed to the corresponding author.

## Supplementary Materials

The following supporting information can be downloaded at: https://www.mdpi.com/article/10.3390/toxics13100808/s1, Text S1. The introduction of kinetic models, isotherm models, and thermodynamic parameters. Table S1. Comparison of literature on modified biochar for phosphate removal. Table S2. Intraparticle diffusion model parameters. Table S3. Thermodynamic parameters of phosphate adsorption at different temperatures. Table S4. Effects of different fertilization measures on the basic physical and chemical properties of tobacco planting soil. Table S5. Soil microbial diversity index for each treatment. Figure S1. Physical photos of tobacco field cultivation (Refs. [62,63,64,65,66,67,68,69] are cited in the Supplementary Materials).
