# Supplementary material for: Efficient Recovery of Phosphorus from Wastewater Using Calcium-Based Modified Biochar: Removal Performance, Adsorption Mechanism, and Resource Utilization
Source: Toxics. 2025 Sep 23;13(10):808. doi: 10.3390/toxics13100808 (PMC12568302; doi:10.3390/toxics13100808)
Supplement: Supplementary file 1 [file toxics-13-00808-s001.zip › toxics-3858421-supplementary.pdf]

Supplementary Material

Efficient recovery of phosphorus from wastewater using calcium-based modified biochar: Removal performance, adsorption mechanism, and resource utilization

Yihe Qin[1,2], Run Yuan[1], Han Li[1], Haiming Huang[1*]

[1] School of Environment and Civil Engineering, Dongguan University of Technology, Dongguan 523808, China; qinyihely@163.com (Y.Q.); qyuanrun@163.com (R.Y.); lihan@dgut.edu.cn (H.L.); huanghaiming0115@163.com (H.H.).

[2] School of Earth System Science, Tianjin University, Weijin Road, Tianjin, 300072, China; qinyihely@163.com (Y.Q.).

[*] Correspondence E-mail: huanghaiming0115@163.com.

12 pages of Supplementary Material, including 1 text, 4 tables, and 1 figure

Text S1. The introduction of kinetic models, isotherm models, and thermodynamic parameters.

Table S1. Comparison of literature on modified biochar for phosphate removal.

Table S2. Intraparticle diffusion model parameters.

Table S3. Thermodynamic parameters of phosphate adsorption at different temperatures.

Table S4. Effects of different fertilization measures on the basic physical and chemical properties of tobacco planting soil.

Table S5. Soil microbial diversity index for each treatment.

Figure S1. Physical photos of tobacco field cultivation.

Text S1. The introduction of kinetic models, isotherm models, and thermodynamic parameters.

1. **Kinetic models:**

The removal process of organic matter during adsorption was analyzed using the pseudo-first-order kinetic model (Equation S1), pseudo-second-order kinetic model (Equation S2), Elovich model (Equation S3), and intraparticle diffusion model (Equation S4), respectively. The specific equations are as follows [1]:

$$q_t = q_e - q_e e^{-K_1 t} \quad \text{Equation S1}$$

$$q_t = \frac{K_2 q_e^2 t}{1+K_2 q_e t} \quad \text{Equation S2}$$

$$q_t = \frac{1}{b}\ln(abt) \quad \text{Equation S3}$$

$$q_t = k_{id} t^{0.5} + C \quad \text{Equation S4}$$

where K_1 (1/min) and K_2 (g/mg/min) are the equilibrium rate constants associated with pseudo-first-order and pseudo-second-order, respectively, a (mg/g/min) is the initial adsorption rate, and b (g/mg) is the desorption constant. The adsorption capacity at equilibrium (q_e, mg/g), at time t (q_t, mg/g), k_{id} (mg/g/h$^{0.5}$) is the intraparticle diffusion rate constant, and C (mg/g) is determined by the thickness of the boundary layer.

2. Isotherm models:

The introduction of Langmuir (Equation S5), Freundlich (Equation S6), Temkin models (Equation S7), R^2 (Equation S8) and root mean square error ($RMSE$) (Equation S9) are as follows [1]:

$$q_e = \frac{q_m K_l C_e}{1+K_l C_e} \quad \text{Equation S5}$$

$$q_e = K_f C_e^n \quad \text{Equation S6}$$

$$q_e = \frac{RT}{B}\ln(AC_e) \quad \text{Equation S7}$$

$$R^2 = 1 - \frac{\sum_{i=1}^{i=n}(\hat{y}_i - y_i)^2}{\sum_{i=1}^{i=n}(\bar{y}_i - y_i)^2} \quad \text{Equation S8}$$

$$RMSE = \sqrt{\frac{\sum_{i=1}^{i=n}(y_i - \hat{y}_i)^2}{n}} \quad \text{Equation S9}$$

where K_l (L/mg) and K_f (mg$^{(1-n)}$·L^n/g) represent the Langmuir bonding term

determined by the affinity and free energy of the adsorption process and the Freundlich affinity coefficient, respectively; q_m (mg/g) indicates the Langmuir maximum adsorption capacity; n (dimensionless) is the Freundlich linearity constant; and B (J·g/mg) and A (L/mg) are the Temkin isotherm constants. C_e is the phosphate concentration at equilibrium time. R and T respectively refer to the gas constant (8.314 J/(mol·K)) and temperature (K), n represents the number of data sets, y_1, y_2…y_n are the actual values, $\overline{y}$ is the average of all actual values, and y_i and $\hat{y}_i$ are the actual value and predicted value for the i-th instance, respectively.

3. Thermodynamic parameters:

Conducting thermodynamic analysis is crucial for the adsorption process. The specific calculation of the theoretical free energy value (Δ G °) is as follows [1]:

$$k_d = \frac{q_e}{C_e} \qquad \text{Equation S10}$$

$$\Delta G^{\circ} = -RTLn(k_d) \qquad \text{Equation S11}$$

where k_d refers to the distribution coefficient, R and T, respectively, refer to the gas constant (8.314 J/(mol·K)) and temperature (K).

The calculation process of enthalpy change (Δ H °) and entropy change (Δ S °) of phosphate is as follows[1]:

$$Ln(k_d) = \frac{\Delta S^{\circ}}{R} - \frac{\Delta H^{\circ}}{RT} \qquad \text{Equation S12}$$

References

[1] Zeng J , Chen D , Zhu J ,et al. Phosphate recovery using activated sludge cyanophycin: Adsorption mechanism and utilization as nitrogen-phosphorus fertilizer[J].Chemical Engineering Journal, 2023, 476(000):11.DOI:10.1016/j.cej.2023.146607.

Table S1

Table S1. Comparison of literature on modified biochar for phosphate removal.

Raw materials	Modification method	Calcination temperature (°C)	Specific surface area (m^2/g)	Adsorption capacity (mg/g)	References
Coffee wastes biochar	Ca and Fe chemical modification	650	78.8	34.5	[1]
Spent coffee grounds biochar	Ca and Mg chemical modification	600	50.61	144.31	[2]
Sawdust feedstock biochar	Ca-Al-La chemical modification	500	—	152.9	[3]
Bamboo scraps biochar	N and Ca co-doped modification	800	129.25	145.47	[4]
Waste wheat straw biochar	Ca and Al chemical modification	450	41.55	218.82	[5]
Municipal sludge	Fe and Ca	600	160.79	1.2	[6]

biochar	modification				
Orange peels biochar	Ca and Zn modification	700	40.71	52.96	[7]
Rice husk biochar	Ca and Si modification	800	35.43	123.3	[8]
Activated sludge-based biochar	Oyster shell chemical modification	800	23.43	129.03	[9]
Vancomycin fermentation residues biochar	Ca and Fe chemical modification	600	76.27	102	[10]

References

[1] SHIN J, RHO H, CHO Y, et al. Functionalization of coffee waste biochars with Ca/Fe layered double hydroxides for enhanced removal and selectivity of phosphate ions: Mechanisms and reusability[J]. Journal of Water Process Engineering, 2025,76: 108123.

[2] CHEN M, LIU Y, PAN J, et al. Low-cost Ca/Mg co-modified biochar for effective phosphorus recovery: Adsorption mechanisms, resourceful utilization, and life cycle assessment[J]. Chemical Engineering Journal, 2024,502: 157993.

[3] CHENG F, WANG Y, FAN Y, et al. Optimized Ca-Al-La modified biochar with rapid and efficient phosphate removal performance and excellent pH stability[J]. Arabian Journal of Chemistry, 2023,16(8): 104880.

[4] YANG Z, ZOU Z, AKHTAR M A, et al. Synergistic effects of N-containing heterocyclic and Ca ligand structures on the phosphorus adsorption of N/Ca co-doped biochar[J]. Journal of Cleaner Production, 2024,485: 144392.

[5] Dan Luo, NAN H, ZHANG Y, et al. Phosphorus recovery from wastewater by Ca-Al layered double hydroxide/biochar as potential agricultural phosphorus for closed-loop phosphorus recycling[J]. Process Safety and Environmental Protection, 2025,194: 1538-1548.

[6] WANG Q, XIAO Y, QI J, et al. A novel strategy for preparing porous Fe/Ca-loaded biochar transformed from municipal sludge towards phosphate removal[J]. Journal of Water Process Engineering, 2024,66: 106109.

[7] CHEN Z, WU Y, HUANG Y, et al. Enhanced adsorption of phosphate on orange peel-based biochar activated by Ca/Zn composite: Adsorption efficiency and mechanisms[J]. Colloids and Surfaces A: Physicochemical and Engineering Aspects, 2022,651: 129728.

[8] CHEN Y, ZHANG R, GAO J, et al. The role of silica in biomass for calcium-modified biochar: Phosphorus removal mechanism and potential as a phosphate fertilizer application[J]. Journal of Environmental Sciences, 2025,158: 242-253.

[9] LI J, CAO L, LI B, et al. Utilization of activated sludge and shell wastes for the preparation of Ca-loaded biochar for phosphate removal and recovery[J]. Journal of Cleaner Production, 2023,382: 135395.

[10] ZHANG M, CHEN Q, ZHANG R, et al. Pyrolysis of Ca/Fe-rich antibiotic fermentation residues into biochars for efficient phosphate removal/recovery from wastewater: Turning hazardous waste to phosphorous fertilizer[J]. Science of The Total Environment, 2023,869: 161732.

Table S2

Table S2. Intraparticle diffusion model parameters.

q_t	K_{id} (mg/(g·min$^{0.5}$))	C(mg/g)	R^2	RMSE
q_{t1}	20.79±1.14	-10.39±2.45	0.992	2.74
q_{t2}	3.60±0.58	116.20±6.22	0.987	0.49
q_{t3}	0.153±0.06	160.95±1.27	0.975	0.14

Table S3

Table S3. Thermodynamic parameters of phosphate adsorption at different temperatures.

T(K)	Kc	ΔG^0 **(kJ/mol)**	ΔS^0 **(J/mol·k)**	ΔH^0**(kJ/mol)**
288	3.5382	-3.0257	46.8336	10.2487
298	4.9875	-3.9813		
308	5.4188	-4.3273		
318	5.3497	-4.4339		

Table S4

Table S4. Effects of different fertilization measures on the basic physical and chemical properties of tobacco planting soil.

Types	pH	Available potassium mg/kg	Available phosphorus mg/kg	Ammonium nitrogen mg/kg	Nitrate nitrogen mg/kg	Total phosphorus mg/kg
BT	7.22±0.08	222.83±5.92	4.204±0.70	0.899±0.01	4.50±0.11	551.6±21.79
NPK	6.57±0.20	261.90±6.44	14.723±0.38	1.512±0.03	14.24±0.43	685.7±22.79
NK	6.90±0.25	231.43±9.06	3.330±0.06	1.723±0.03	13.30±0.54	531.9±16.06
BP	7.68±0.20	319.00±0.77	21.438±0.63	2.410±0.08	24.16±0.65	767.3±26.46

Table S5

Table S5. Soil microbial diversity index for each treatment.

Types	chao1	Shannon	Simpson
BT	2693.79±101.34	10.23±0.09	0.998±0
NPK	2811.16±288.25	10.24±0.18	$0.998\pm4.71e^{-4}$
NK	2672.06±93.78	10.03±0.07	0.997±0
BP	2890.78±54.56	10.39±0.06	$0.998\pm4.71e^{-4}$

Figure S1

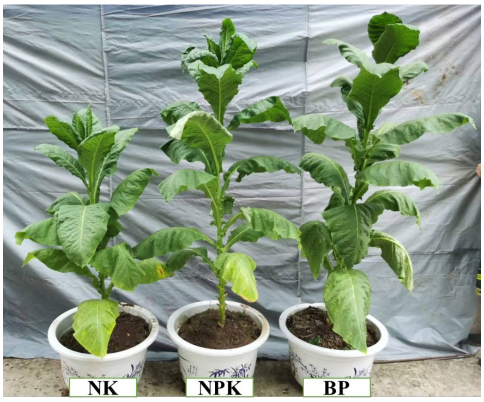

Figure S1: Physical photos of tobacco field cultivation.
